# Guideline Concordance of Treatment and Outcomes Among Adult Non-Hodgkin Lymphoma Patients in Sub-Saharan Africa: A Multinational, Population-Based Cohort

**DOI:** 10.1093/oncolo/oyad157

**Published:** 2023-06-27

**Authors:** Nikolaus Christian Simon Mezger, Lucia Hämmerl, Mirko Griesel, Tobias Paul Seraphin, Yvonne Walburga Joko-Fru, Jana Feuchtner, Annelle Zietsman, Jean-Félix Péko, Fisihatsion Tadesse, Nathan Gyabi Buziba, Henry Wabinga, Mary Nyanchama, Eric Chokunonga, Mamadou Kéita, Guy N’da, Cesaltina Ferreira Lorenzoni, Marie-Thérèse Akele-Akpo, Jörg Michael Mezger, Mascha Binder, Biying Liu, Marcus Bauer, Oliver Henke, Ahmedin Jemal, Eva Johanna Kantelhardt

**Affiliations:** Global Health Working Group, Institute of Medical Epidemiology, Biometrics and Informatics, Martin-Luther-University Halle-Wittenberg, Halle, Germany; Global Health Working Group, Institute of Medical Epidemiology, Biometrics and Informatics, Martin-Luther-University Halle-Wittenberg, Halle, Germany; Global Health Working Group, Institute of Medical Epidemiology, Biometrics and Informatics, Martin-Luther-University Halle-Wittenberg, Halle, Germany; Global Health Working Group, Institute of Medical Epidemiology, Biometrics and Informatics, Martin-Luther-University Halle-Wittenberg, Halle, Germany; African Cancer Registry Network, Oxford, UK; Nuffield Department of Population Health, University of Oxford, Oxford, UK; Global Health Working Group, Institute of Medical Epidemiology, Biometrics and Informatics, Martin-Luther-University Halle-Wittenberg, Halle, Germany; African Cancer Registry Network, Oxford, UK; Dr AB May Cancer Care Centre, Windhoek, Namibia; African Cancer Registry Network, Oxford, UK; Registre des cancers de Brazzaville, Brazzaville, Republic of the Congo; African Cancer Registry Network, Oxford, UK; Division of Hematology, Department of Internal Medicine, University and Black Lion Hospital, Addis Ababa, Ethiopia; African Cancer Registry Network, Oxford, UK; Eldoret Cancer Registry, School of Medicine, Moi University, Eldoret, Kenya; African Cancer Registry Network, Oxford, UK; Kampala Cancer Registry, Makerere University School of Medicine, Kampala, Uganda; African Cancer Registry Network, Oxford, UK; National Cancer Registry, Kenya Medical Research Institute, Nairobi, Kenya; African Cancer Registry Network, Oxford, UK; Zimbabwe National Cancer Registry, Harare, Zimbabwe; African Cancer Registry Network, Oxford, UK; Service du Laboratoire d’Anatomie et Cytologie Pathologique, Bamako, Mali; CHU du point G , Bamako, Mali; African Cancer Registry Network, Oxford, UK; Registre des cancers d’Abidjan, Abidjan, Côte d’Ivoire; African Cancer Registry Network, Oxford, UK; Departamento de Patologia, Faculdade de Medicina, Universidade Eduardo Mondlane, Hospital Central de Maputo, Mozambique; Registo de Cancro, Ministério da Saúde, Maputo, Mozambique; African Cancer Registry Network, Oxford, UK; Département d’anatomo-pathologie, Faculté des Sciences de la Santé, Cotonou, Benin; Albert-Ludwig University of Freiburg, Germany; Department of Internal Medicine IV, Oncology/Hematology, Martin-Luther-University Halle-Wittenberg, Halle, Germany; African Cancer Registry Network, Oxford, UK; Institute of Pathology, Martin-Luther-University Halle-Wittenberg, Halle, Germany; Section Global Health, Institute for Public Health and Hygiene, University Hospital Bonn, Germany; Surveillance and Health Equity Science, American Cancer Society, Atlanta, USA; Global Health Working Group, Institute of Medical Epidemiology, Biometrics and Informatics, Martin-Luther-University Halle-Wittenberg, Halle, Germany; Department of Gynaecology, Martin-Luther-University Halle-Wittenberg, Halle, Germany

## Abstract

**Background:**

Although non-Hodgkin lymphoma (NHL) is the 6th most common malignancy in Sub-Saharan Africa (SSA), little is known about its management and outcome. Herein, we examined treatment patterns and survival among NHL patients.

**Methods:**

We obtained a random sample of adult patients diagnosed between 2011 and 2015 from 11 population-based cancer registries in 10 SSA countries. Descriptive statistics for lymphoma-directed therapy (LDT) and degree of concordance with National Comprehensive Cancer Network (NCCN) guidelines were calculated, and survival rates were estimated.

**Findings:**

Of 516 patients included in the study, sub-classification was available for 42.1% (121 high-grade and 64 low-grade B-cell lymphoma, 15 T-cell lymphoma and 17 otherwise sub-classified NHL), whilst the remaining 57.9% were unclassified. Any LDT was identified for 195 of all patients (37.8%). NCCN guideline-recommended treatment was initiated in 21 patients. This corresponds to 4.1% of all 516 patients, and to 11.7% of 180 patients with sub-classified B-cell lymphoma and NCCN guidelines available. Deviations from guideline-recommended treatment were initiated in another 49 (9.5% of 516, 27.2% of 180). By registry, the proportion of all patients receiving guideline-concordant LDT ranged from 30.8% in Namibia to 0% in Maputo and Bamako. Concordance with treatment recommendations was not assessable in 75.1% of patients (records not traced (43.2%), traced but no sub-classification identified (27.8%), traced but no guidelines available (4.1%)). By registry, diagnostic work-up was in part importantly limited, thus impeding guideline evaluation significantly. Overall 1-year survival was 61.2% (95%CI 55.3%-67.1%). Poor ECOG performance status, advanced stage, less than 5 cycles and absence of chemo (immuno-) therapy were associated with unfavorable survival, while HIV status, age, and gender did not impact survival. In diffuse large B-cell lymphoma, initiation of guideline-concordant treatment was associated with favorable survival.

**Interpretation:**

This study shows that a majority of NHL patients in SSA are untreated or undertreated, resulting in unfavorable survival. Investments in enhanced diagnostic services, provision of chemo(immuno-)therapy and supportive care will likely improve outcomes in the region.

## Teaser

Non-Hodgkin lymphoma (NHL) is the sixth most common type of malignant neoplasia in Sub-Saharan Africa; however, resources for cancer care are limited. This article assesses the application of NHL treatment according to NCCN harmonized guidelines in this region.

**Implications for Practice**

Although advances in care have tremendously improved non-Hodgkin lymphoma (NHL) outcomes, disparities in uptake of treatment still confine survival across the globe. While NHL is a common disease in Sub-Saharan Africa, little is known about its treatment and survival. Our multinational, population-based study aimed to assess the current quality of care and survival in 10 countries. Patients across the region presented at late stages, with poor ECOG performance status, and lacked subtyping. Absence of any therapy was identified in some 3 in 5 patients, and non-guideline-concordant therapy in 6 of 7, with all factors associated with unfavorable survival. Our study shows that many NHL patients are unable to access high-quality diagnostic and treatment services, providing a baseline for targeted investments. With regard to clinical practice, we underline the importance of NHL grading and subtyping, patient-centered treatment mindful of possible side effects, and relevance of therapy completion.

## Introduction

Non-Hodgkin lymphoma (NHL) is the 6th most common type of malignant neoplasia in Sub-Saharan Africa (SSA).^1,2^ Incidence is continuously rising and by 2040 the number of new cases per year is expected to nearly double to more than 60 000.^3-5^ Many subtypes of NHL are treatable with good outcomes, with a 5-year survival rate of 73.2% for patients in the United States.^6^ In SSA, however, resources for cancer care are limited.^7-10^ Therefore, the National Comprehensive Cancer Network (NCCN) developed Harmonized Guidelines on a variety of B-cell lymphoma subtypes for resource-stratified use in the region.^11^ In this context, identification of NHL subtype is crucial for specific therapy, however, a high frequency of unclassified lymphoma has been reported across the region.^8-10^

Previous studies on NHL treatment patterns in SSA were hospital-based studies, with high proportion of late-stage and aggressive diseases,^8,10,12-16^ limited treatment options, and poor survival.^10,17-22^ The aim of our study was to assess the application of NHL treatment according to NCCN harmonized guidelines in this region and to identify factors influencing survival using a multi-national, real-world cohort within the African Cancer Registry Network (AFCRN, https://afcrn.org).

## Methods

### Study Setting

**Figure 1.**
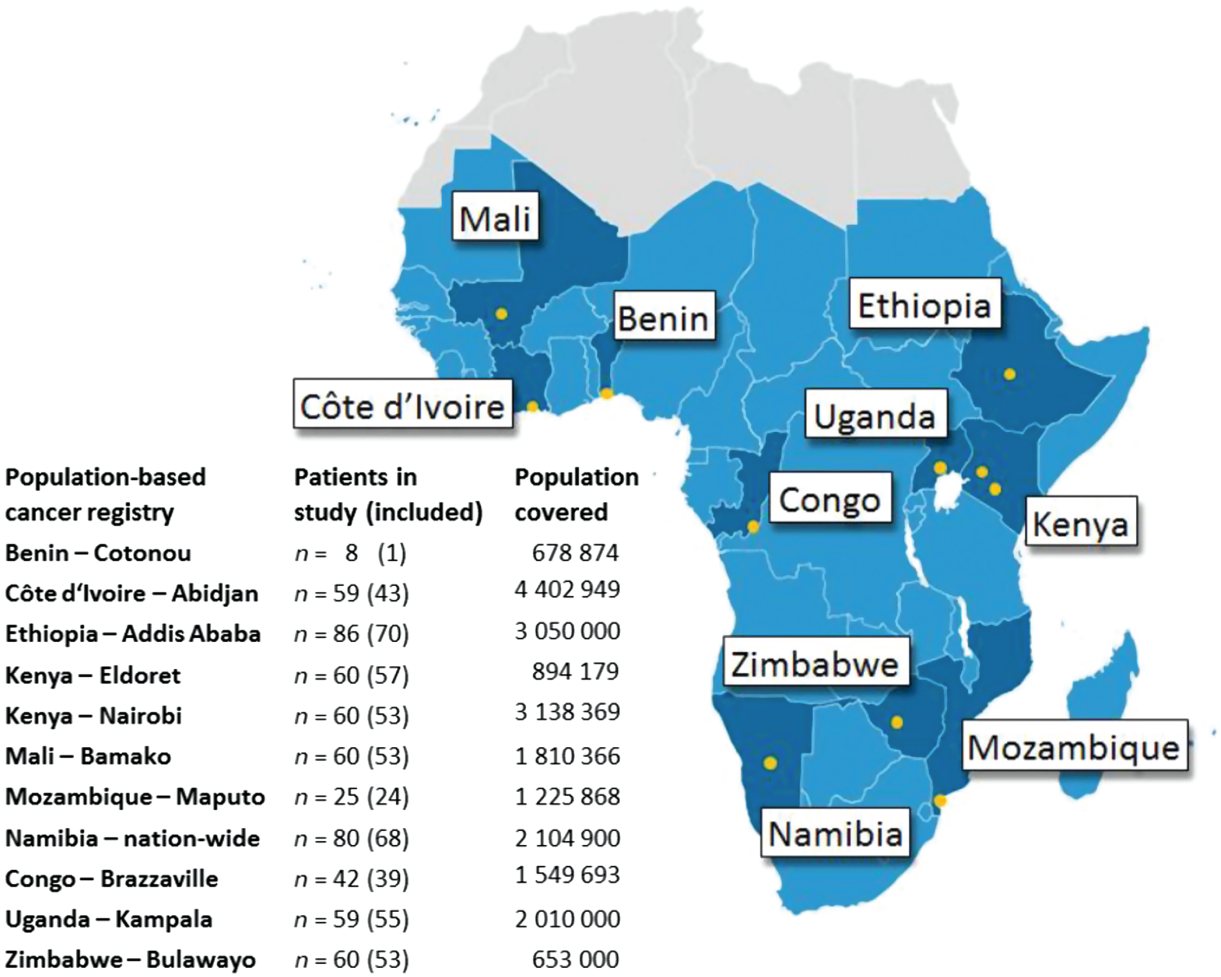
Map of Sub-Saharan Africa.^24,25^ Countries and cities of participating population-based cancer registries are highlighted. On the left, the numbers included in the random sample are shown along with the covered population in the registry area. For details see also Supplementary Table S2.

In 2014, AFCRN coordinated 23 regional population-based cancer registries (PBCRs) as International Agency for Research on Cancer’s regional hub in SSA.^23^ Of these, 11 registries in 10 countries consented to serve as study centers, covering a population of roughly 21.5 million (Fig. 1). We included NHL patients aged 15 and above with B-cell and T-cell lymphoma as well as unclassified lymphoma (International Classification of Diseases-10 codes C82–C96 and C96) and diagnosed between 2011 and 2015. Hodgkin lymphoma and pediatric lymphoma aged 14 and below were not included. Power was calculated for the entire cohort but not for individual sites: A minimal sample size of 404 patients produces a 2-sided 95% CI with a width equal to 0.1 when the sample proportion of patients with adequate care is 0.500. We assumed a drop-out rate of 33% and therefore aimed for 600 patients. Of 1068 patients available, a study population of 599 patients (56.1%) was thus selected at random.

### Data Collection

As previously described in detail, registry staff continuously retrieve information on demographics, diagnosis including NHL subtype, and vital status from hospital records.^24^ Occasionally, data on treatment modalities (eg, chemotherapy yes/no) are collected. To complement PBCR routine data, clinical records were re-evaluated to collect information on patterns of care. Lymphoma morphology registered was verified and amended by assessing pathology reports, and, in the absence of definitive pathological diagnoses, those noted in clinical records were used.^24^ Stage was assessed in line with Lugano and Binet classifications.^26,27^ When the stage had not been assigned in records, it was considered less advanced if no suggestion of disseminated nodal or extranodal involvement was found. Vital status was assessed by follow-up calls. Patients were considered “traced” if information beyond PBCR data (eg, detailed information on clinical diagnostics (such as ECOG performance status (PS) or HIV status) and/or lymphoma-directed treatment (such as chemotherapy regimen administered or radiotherapy) and/or survival status) was obtained from hospital records and/or follow-up calls. Patients were considered “not traced” if no information beyond PBCR data were available. Follow-up was open for 7 years until April 31, 2018.

### Therapy Evaluation

For NHL subtypes with NCCN Harmonized Guidelines for SSA^11^ available, we established an evaluation scheme assessing completion of first-line therapy and adherence to guidelines. For therapy evaluation, patients were allocated to 3 groups: sub-classified NHL with guidelines available, sub-classified NHL without guidelines available, and unclassified NHL. NCCN Harmonized Guidelines for SSA were available for diffuse large B-cell lymphoma (DLBCL), chronic lymphocytic leukemia/small lymphocytic lymphoma (CLL/SLL), Burkitt (BL), follicular (FL), marginal zone, and lymphoplasmacytic lymphoma. For these subtypes, “guideline concordance” was defined as NCCN’s harmonized “generally available standard of care.” “Deviation from guidelines” was defined, again according to NCCN, as “regional options that may be considered when availability precludes standard of care.” Non-guideline concordant lymphoma-directed therapy (LDT) was defined “any other therapy.” As an example, for DLBCL, NCCN recommends rituximab (R) + cyclophosphamide, vincristine, doxorubicin, and prednisone (CHOP). Deviation from guidelines in DLBCL was thus defined as CHOP without rituximab. Other chemotherapy regimens were labeled as any other therapy. Concerning guideline-concordant therapy *completion* for DLBCL, at least 5 cycles of RCHOP or 3 cycles of RCHOP + radiotherapy in stage I or II were necessary for therapy to be considered complete. For completion of guideline-deviating therapy in DLBCL, the same number of cycles for CHOP was necessary. Concerning guideline concordance of treatment for indolent NHL, NCCN guidelines allow for a variety of chemo(immuno-)therapeutic agents. However, due to the heterogeneous nature of eg, CLL/SLL and FL, NCCN does not specify a minimum number of cycles. Thus, any number of cycles of chemo(immuno-)therapy was accepted regarding the completeness of guideline-concordant therapy (for details on therapy evaluation see Supplementary Table S1). Patients with clinical records traced, but without any information on LDT were labeled as “no therapy.” For presentation of therapy evaluation, patients not traced without PBCR information on LDT were grouped separately. Both for subtypes without guidelines available and for unclassified NHL, application of guidelines was not feasible. We differentiated between polychemo(immuno-)therapy (PCT) vs. “any other therapy” vs. “no therapy,” considering sole radiotherapy without chemo(immuno-)therapy as “any other therapy.” Similarly, we labeled sole splenectomy and other operations in stage I lymphoma as “any other therapy,” but regarded all other operations as supportive care and therefore defined these as “no therapy.”

### Statistical Analysis

For statistical analysis, IBM SPSS Statistics (version 25) was used. For longitudinal data, Kaplan-Meier’s method and multivariable Cox proportional hazard model were used. First, we assessed for the condition of “missing at random” (uninformative censoring) by performing reverse Kaplan-Meier’s analysis. We then restricted the analysis to patients with the survival of at least 1 month to allow time for initiation of therapy and to account for bias from missing treatment through early death. Kaplan-Meier’s method accounted for further loss to follow up. For survival analysis, we grouped patients traced without indication of LDT and patients not traced, assuming that patients not traced despite our efforts did not receive any LDT. We estimated simple and multivariable hazard ratios (HR), and computed 1- to 3-year age-standardized overall survival using the “popEpi” package for R software, while adopting Corazziari et al’s ICSS 1 age standard.^28^

### Ethical Consideration

The study protocol was approved by the AFCRN research committee (March 2, 2016) and the Martin-Luther-University, Halle Ethical Review Board, and it was in line with the Declaration of Helsinki. Anonymized secondary data were collected from each participating registry under existing regulations and national laws of the respective registries.

### Role of the Funding Source

Funders had no role in study design, collection analysis, and interpretation of data, in writing of the report, and in decision to submit the paper for publication.

## Results

**Figure 2.**
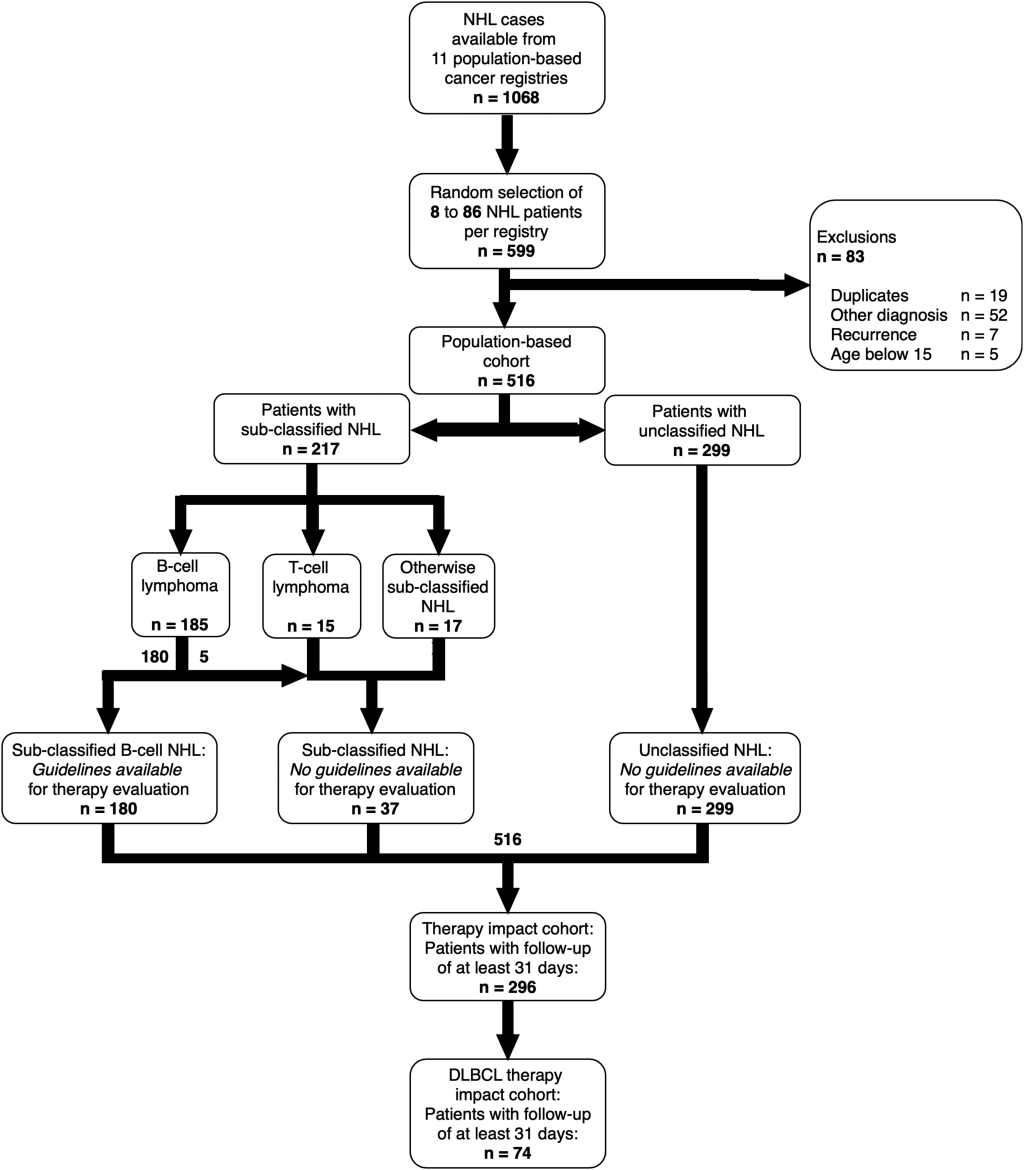
Flow chart of the study population. NHL, non-Hodgkin lymphoma; DLBCL, diffuse large B-cell lymphoma.

Of 599 patients, 516 patients were included (Fig. 2). A total of 83 patients had to be excluded due to duplicates, other diagnoses, recurrence, or not meeting the age inclusion criteria. Additional information, eg, on treatment and/or survival was obtained for 293 patients (“traced,” 56.8%, Supplementary Table S2).

### Baseline and Diagnostic Characteristics

Patient characteristics have been published elsewhere in detail.^24^ Median age was 45 years and 43.4% of patients were female. ECOG PS of 2 or worse was documented in 61.4%, and 79.3% presented with B symptoms. Advanced stage, defined as Lugano stages III and IV and as Binet C for CLL/SLL, was diagnosed in 73.0%. Of 154 tested patients, 63.0% were HIV positive (Supplementary Table S3). In 85.3% combined antiretroviral therapy had been initiated prior to diagnosis of NHL. Sub-classification was documented in 217 patients (42.1) while 299 NHL (57.9%) remained unclassified. By registry, proportion of sub-classified NHL ranged from 94.1% in Namibia to 8.3% in Maputo.^24^ Of all sub-classified lymphoma, 121 were high-grade (55.8%) and 64 low-grade B-cell lymphoma (29.5%), 15 T-cell lymphoma (6.9%), and 17 otherwise sub-classified NHL (7.8%) (Supplementary Table S4).

### Therapy

Any systemic therapy was documented in 187 of all 516 patients (36.2%). For these, first-line chemo(immuno-)therapy consisted of CHOP (-related) and cyclophosphamide, vincristine, and prednisone (COP) (-related) protocols in 62.0% and 10.7%, respectively. Rituximab was the only immunotherapy agent identified and administered in 20 of 187 (10.7%). Patients received a median of 6 cycles of first-line systemic therapy (interquartile range: 3–6 cycles). Among all 83 patients receiving a minimum of 6 cycles of systemic therapy, 49 had sub-classified NHL and 34 unclassified NHL. Overall, 2 patients received second-line systemic therapy. Of the 195 patients with any LDT initiated (37.8%), radiotherapy was identified in 34 cases, and lymphoma-directed surgery in 28 (Table 1). For details, see Supplementary Table S5.

**Table 1.** Treatment modalities in the population-based cohort (*n* = 516).

						
Chemo(immuno-)therapy regimen	Patients (*n*)	% of all receiving systemic therapy	Cycles applied	Patients (*n*)	% of therapy evaluation cohort	Cycles applied, median
CHOP and similar	116	62	5 or more	71	38	6
			4 or less	34	18.2	2
			Unknown # of cycles	11	5.9	n/a
COP and similar	20	10.7	5 or more	10	5.3	6
			4 or less	9	4.8	2
			Unknown # of cycles	1	0.5	n/a
Other polychemo(immuno-)therapy regimen	8	4.3	5 or more	3	1.6	7
			4 or less	5	2.7	3
Monotherapy	15	8	5 or more	3	1.6	6
			4 or less	5	2.7	4
			Unknown # of cycles	7	3.7	n/a
Unknown regimen	28	15	5 or more	5	2.7	6
			4 or less	2	1.1	3
			Unknown # of cycles	21	11.2	n/a
Any systemic therapy	187	100	5 or more	92	49.2	6
			4 or less	55	29.4	2
			Unknown # of cycles	40	21.4	n/a
Radiotherapy dose applied		% of all receiving radiotherapy				
Thirty gray or more	17	48.1				
Less than 30 gray	7	22.2				
Unknown dose	10	29.6				
Any radiotherapy	34	100				
Surgery type		% of all receiving surgery				
Splenectomy and stage I lymphnode resection	5	17.9				
Other surgery (diagnostic/palliative/unspecified)	23	82.1				
Any lymphoma-directed surgery (including diagnostic surgery, excluding biopsies)	28	100				
Any lymphoma-directed therapy	195	37.8 (of population-based cohort, n = 516				

Abbreviations: CHOP, cyclophosphamide, doxorubicin, vincristine, prednisone; COP, cyclophosphamide, vincristine, prednisone.

### Guideline Concordance

**Figure 3.**
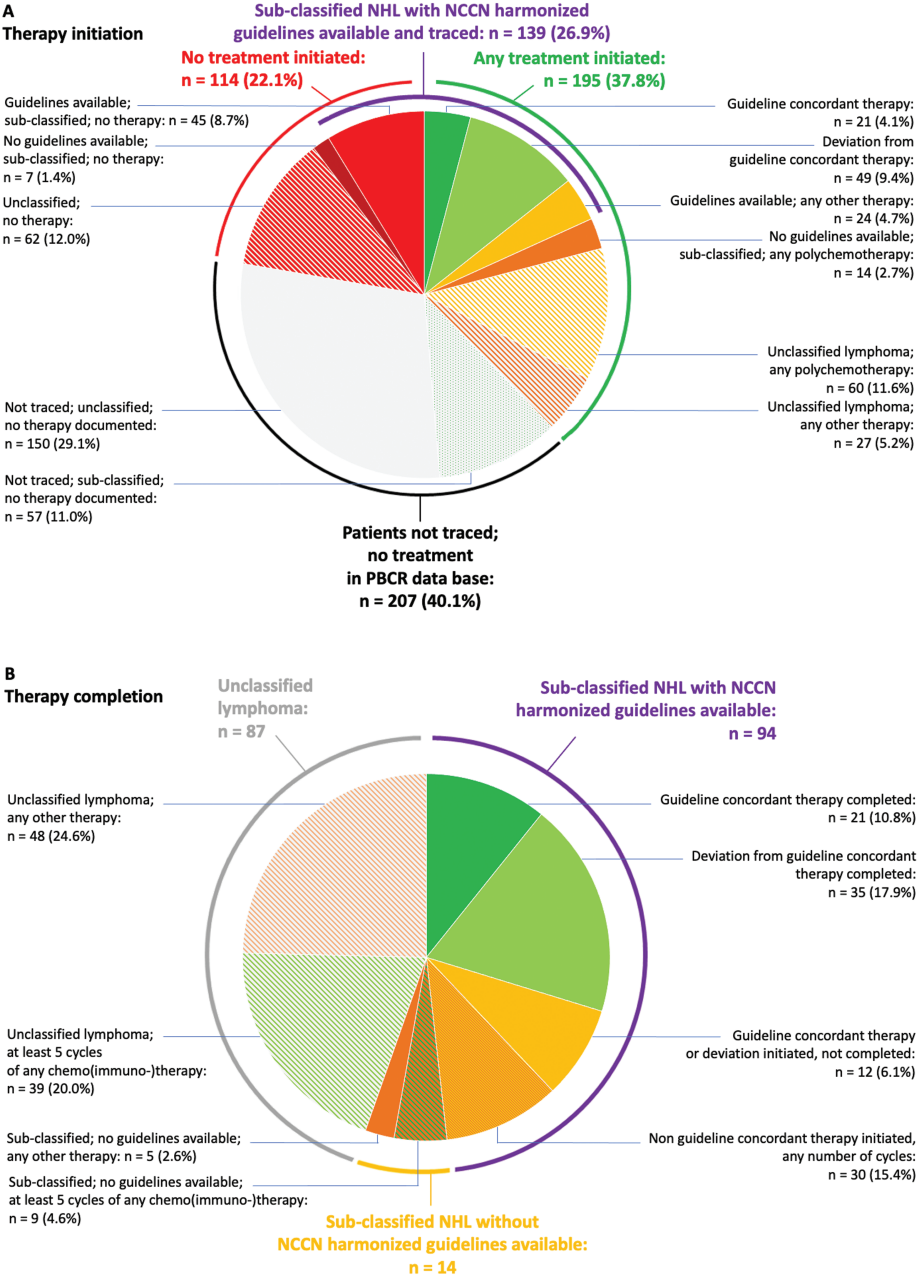
Evaluation of guideline concordance. (**A**) Depicts evaluation of therapy *initiation* in the population-based cohort (*n* = 516). Percentages refer to the proportion of all patients in cohort. (**B**) Depicts evaluation of therapy *completion* in all patients with any treatment documented (*n* = 195 (37.8% of total cohort)). The groups marked in green depict patients completing at least 5 cycles of chemo(immuno-)therapy. Percentages refer to proportion of all patients with any treatment documented. Evaluation refers to “therapy evaluation scheme” in Supplementary Table S1. PBCR, population-based cancer registry.

Of all 516 patients, 180 patients with sub-classified NHL and guidelines available were eligible for therapy evaluation. Namely, patients diagnosed with DLBCL (48.8% of all 217 sub-classified NHL), CLL/SLL (18.8%), BL (6.0%), FL (5.5%), marginal zone (3.2%), and lymphoplasmacytic lymphoma (1.0%) were evaluated with respect to concordance with the NCCN guidelines harmonized for SSA.^11^ Of these 180 cases, we found both initiation and completion of guideline-recommended treatment for 21 patients (11.7%) (Fig. 3A and 3B). Initiation of guideline-deviating therapy was found for another 49 (27.2%), of which 35 (19.4%) managed to complete respective therapies. No therapy could be identified for 86 of 180 cases (47.8%, including patients not traced). For the remaining 37 patients with sub-classified NHL, predominantly T-cell and otherwise sub-classified NHL, no harmonized guidelines were available. Further, no guidelines were available for the 299 patients with unclassified NHL.

### Disparities Within and Between Registries

**Figure 4.**
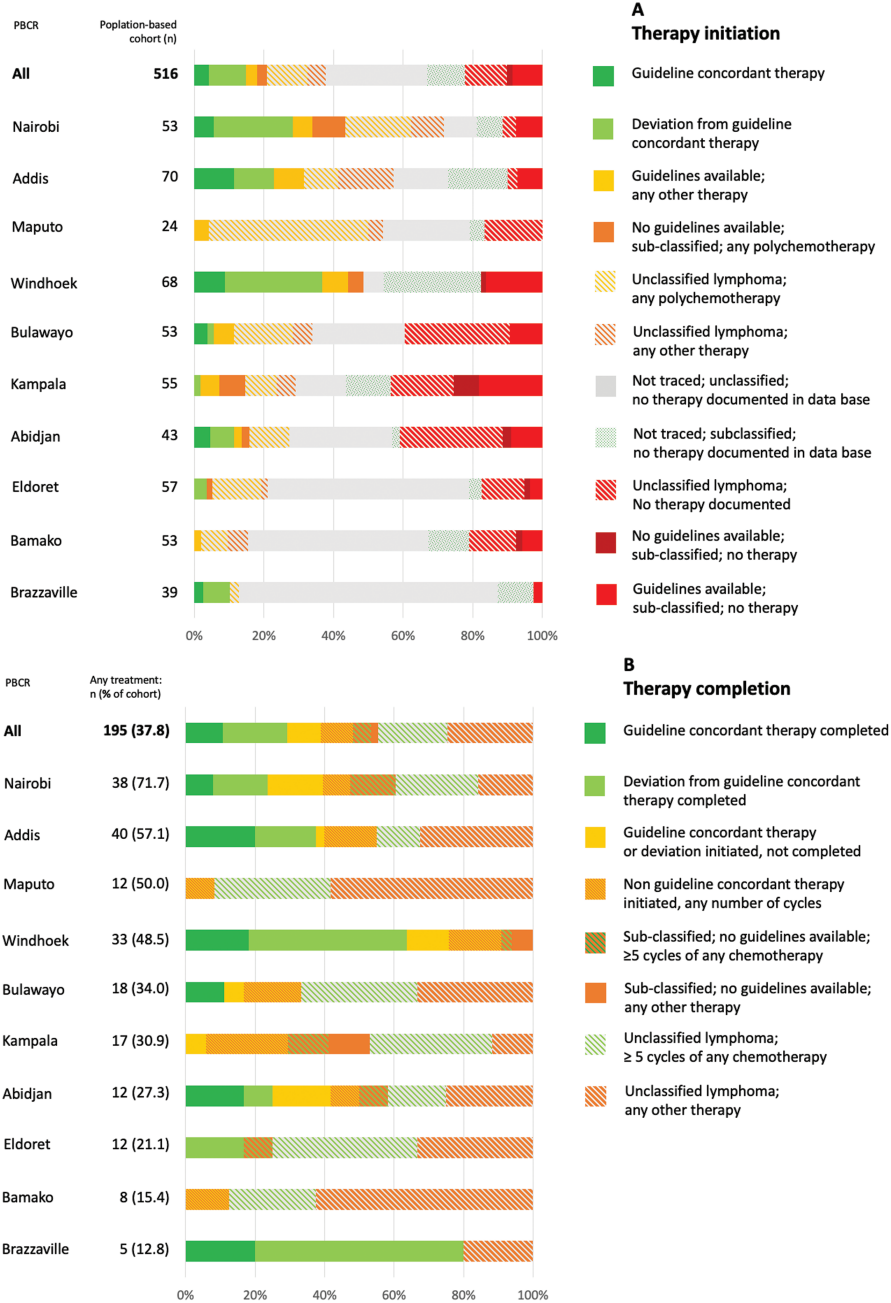
Stratification of evaluation of guideline concordance by population-based cancer registries. (**A**) Depicts evaluation of therapy *initiation* within the population-based cohort (*n* = 516). Percentages refer to proportion of all patients in respective population-based cancer registries. (**B**) Depicts evaluation of therapy *completion* among all patients with any treatment documented (*n* = 195 (37.8% of total cohort)). Percentages refer to proportion of all patients with any treatment documented in respective population-based cancer registries. Evaluation refers to “Therapy evaluation scheme” in Supplementary Table S1. Cotonou was excluded from figure due to small patient number (*n* = 1). PBCR, population-based cancer registry.

Within and between the PBCR cohorts, we found huge disparities in therapy initiation, ranging from patients without any treatment to patients treated in concordance with guidelines. For example, 11.6% of patients in Abidjan initiated guideline-concordant therapy or a deviation thereof, while in 72.1% no treatment was documented. Similarly, in Bamako and Brazzaville only 15.4% and 12.8% had any treatment documented, respectively (Fig. 4A). The largest proportion of patients with any treatment initiated was found in Nairobi (71.7%) followed by Addis Ababa (57.1%). In Namibia, the largest proportion of patients completed therapy concordantly with guidelines (30.8%), for Maputo and Bamako, none were treated in concordance with guidelines—with only 11 sub-classified NHL cases in Bamako (20.8%) and 2 cases in Maputo (8.3%) (Fig. 4B). Radiotherapy was identified in patients from 4 registries only, Addis Ababa, Kampala, Nairobi, and Namibia.

### Survival

**Figure 5.**
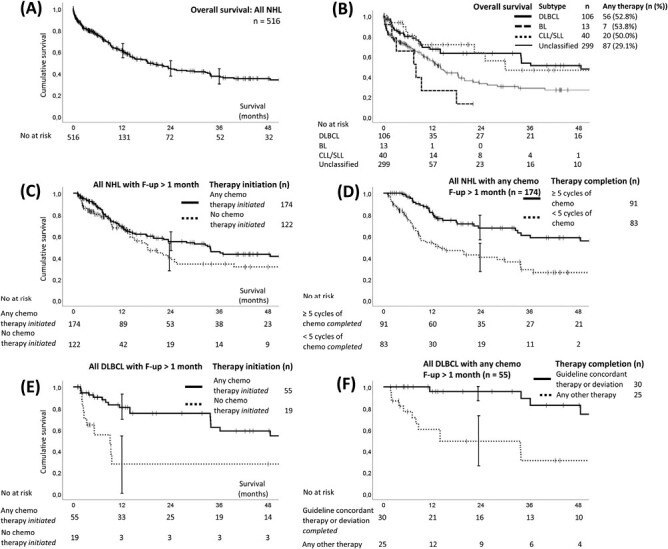
Survival by Kaplan-Meier estimates. (**A**) Overall survival of population-based cohort (*n* = 516); 95% CI indicated for 12, 24, and 36 months. (**B**) Overall survival of population-based cohort stratified by different subtypes and unclassified lymphoma. (**C**) Survival of population-based cohort with at least 1 month of survival (*n* = 296) with respect to therapy initiation and (**D**) those surviving at least 1 month that initiated any chemotherapy (*n* = 174), with respect to completion of chemo(immuno-)therapy cycles. (**E**) Survival of DLBCL with at least 1 month of survival (*n* = 74) with respect to therapy initiation and (**F)** DLBCL patients surviving at least 1 month that received any chemo(immuno-)therapy (*n* = 55) with respect to therapy completion concording with NCCN guidelines harmonized for Sub-Saharan Africa. No, Number; DLBCL, diffuse large B-cell lymphoma; BL, Burkitt lymphoma; CLL/SLL, chronic lymphocytic leukemia/small lymphocytic lymphoma; F-up, follow-up.

Any follow-up information was available for 384 patients. For all patients, median follow-up and survival were 6 and 20 months, respectively. Observed 1- and 3-year overall survival (OS) was 61.2% (95% CI, 55.3%-67.1%) and 37.2% (30.5%-43.9%) (Fig. 5A), respectively, varying substantially between the different PBCR areas: 1-year-OS was highest for patients in Addis Ababa (76.3%) and worst for patients in Bulawayo (37.5%) (Supplementary Table S6). The 1-and 3-year age-standardized overall survival was 62.3% (95%CI, 52.9%-70.4%) and 32.9% (22.1%-44.2%), respectively. As for median survival of subtypes, we found 48 months in DLBCL (*n* = 110), 29 months in CLL/SLL (*n* = 40), 8 months in BL (*n* = 13), 9 months in FL (*n* = 12), and 15 months in unclassified lymphoma (Fig. 5B). Differences in survival with respect to any therapy initiation in all NHL were rather small (Fig. 5C), but better survival was found in patients completing at least 5 cycles of chemo(immuno-)therapy (Fig. 5D). In DLBCL, both any therapy initiation as well as completion of guideline-recommended treatment were associated with better survival (Fig. 5E and 5F). Kaplan-Meier estimates for clinical characteristics and further association of guideline-concordant treatment with improved survival are shown in Supplementary Fig. S1.

### Factors Associated With Outcome

In unadjusted Cox proportional hazards modeling, mortality of the cohort (follow-up at least 30 days, *n* = 296) was associated with ECOG PS, presence of B symptoms, missing assessment of B symptoms, advanced or missing stage, and somewhat associated with lack of subtype. Mortality was also associated with receipt of less than 5 cycles of any chemo(immuno-)therapy and lack of treatment. For DLBCL (*n* = 74), we found mortality associated with age of 60 and older, absent staging, and lack of guideline-concordant therapy or absence of any therapy. Notably, for neither cohort HIV status was associated with mortality (Supplementary Table S7).

**Figure 6.**
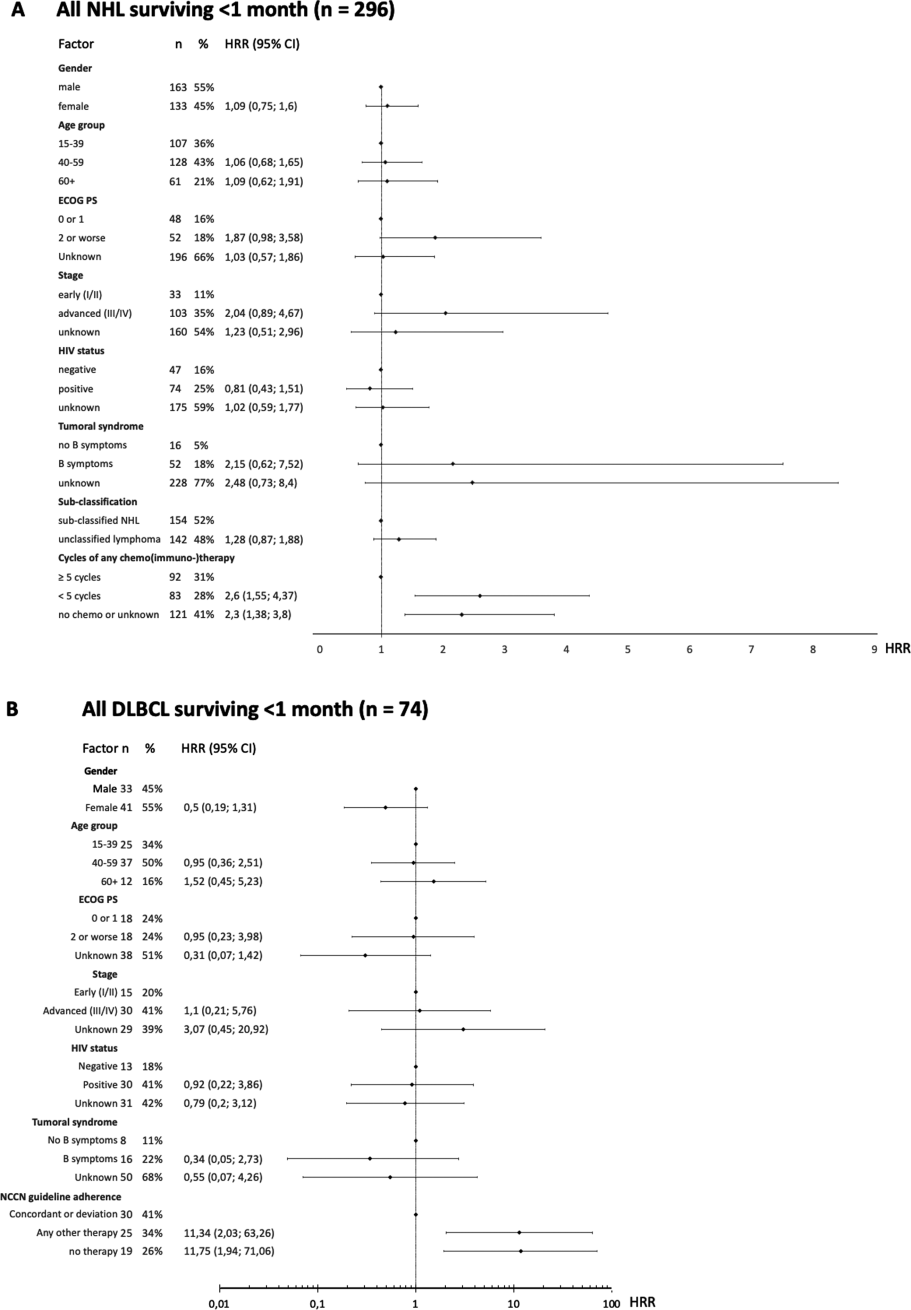
Results of multivariable Cox regression analysis for risk of early death. **A:** All NHL in the population-based cohort with at least 1 month of survival (*n* = 296). **B:** All DLBCL in the population-based cohort with at least 1 month of survival (*n* = 74). HRR, hazard rate ratio.

In adjusted Cox proportional hazards modeling controlling for selected parameters in all NHL patients, worse survival remained (somewhat) associated with worse ECOG PS, advanced stage, B symptoms, less than 5 cycles of any chemo(immuno-)therapy, and absence of any therapy (Fig. 6A). For DLBCL patients only, absent staging and initiation of therapy other than guideline-recommended and absence of any therapy remained (somewhat) associated with worse survival in multivariate Cox regression (Fig. 6B).

Reverse Kaplan-Meier analysis suggested that in all NHL patients as well as in the DLBCL cohort, some covariates had a similar pattern of censoring over time: for sex, site involved, and HIV status, censoring appeared at random. NHL patients with ECOG PS of 1 or better versus others, early-stage versus others, lack of B symptoms, sub-classified NHL as well as completion of at least 5 cycles of any chemotherapy versus others, had less censoring. For DLBCL patients, ECOG PS of 1 or better, any staging, and initiation of guideline-concordant therapy equally had less censoring.

## Discussion

This study represents, to our knowledge, the first population-based multinational investigation on treatment and survival in adult non-Hodgkin lymphoma patients in Sub-Saharan Africa. Our objective was to evaluate guideline-concordance of therapy and survival in real-world patients. The main results of our study were: (1) The proportion of patients treated was low and guideline-concordant therapy was initiated in very few patients. (2) Survival of our study population was poor, while guideline-concordant treatment was associated with improved outcomes. (3) Treatment and survival of NHL patients varied considerably within and between the population-based cancer registries included.

(1) A concerning finding is the small share of NHL patients that received guideline-concordant care. Roughly summarized, NCCN Harmonized Guidelines for SSA recommend intensified chemotherapy regimen plus rituximab for the predominant aggressive subtypes such as DLBCL and BL as well as for advanced FL and MZL, and monotherapy for CLL/SLL.^11^ However, only 13.1% of patients in our population-based cohort initiated guideline-concordant treatment or therapy with some deviation. As reported previously by our group in detail, one important factor attributing to this strikingly low proportion is the absence of sub-classification in more than half of patients (57.9%) and hence failure to apply guideline-concordant therapy.^24^ Our results stress the importance of diagnostic work-up in NHL. Uniform treatment approaches disregarding subtype of lymphoma appear common in the region, eg, administration of oral polychemotherapy or (R-)CHOP for any NHL.^10,22,29^ Only in recent years, multiple hospital-based studies have shed more light on feasibility of grade- and subtype-directed treatment approaches in SSA, eg, on AIDS-related DLBCL,^30^ aggressive B- and T-cell lymphoma,^20^ BL,^18^ and HIV-associated aggressive NHL.^31^ We suggest that in case of further amendment of NCCN Harmonized Guidelines, recommendations for treatment of high- and low-grade lymphoma may be considered when further subtyping is not feasible.
  Another reason may be the lack of certain treatments even when sub-classification of NHL is available. Almost all patients in our cohort received CHOP- (73.0%) or COP-based (12.6%) regimens. An important factor contributing to absence of differentiated treatment may be cost and availability of chemotherapy agents (eg, highly effective bendamustine for MZL and CLL/SLL^11^). In high-income countries, the introduction of rituximab has led to unprecedented rates of long-term cure and control of B-cell lymphoma.^32,33^ CD20 antibodies are included in NCCN Harmonized Guidelines for several B-cell lymphoma subtypes,^11^ and cost of biosimilars tends to be lower than rituximab.^7^ However, they seemed hardly available in most SSA settings at the time^7,22^ though recently proven safe, efficient,^17^ and cost-effective for Malawi.^34^ In our cohort, the majority of the 20 patients receiving rituximab came from Namibia, a middle-income country where public health insurance started covering the drug in 2013. To improve evidence-based treatment for predominantly aggressive lymphoma of B-cell lineage in SSA, health systems across SSA should increase efforts to procure and provide a wider range of systemic therapy agents at low cost, first and foremost rituximab or its biosimilars. Inclusion of not least CD20 antibodies in universal health coverage could leverage provision of adequate care for patients in the region. A fourth reason for low proportion of guideline-concordant care is the lack of NCCN Harmonized Guidelines for T-cell NHL and other rare entities such as plasmablastic and mantle cell lymphoma (17.1% of all sub-classified NHL).^11^ More importantly, fifth, no treatment was documented in 114 of 297 patients traced (39.4%), and despite thorough investigation, another 217 of the 516 patients could not be traced (42.1%). In a worst-case scenario, where all untraced patients received no therapy, the share of patients without any lymphoma-directed treatment would amount to 62.2%.
(2) Overall survival in our study was poor (61.2% one-year survival), but slightly higher than outcomes reported by hospital-based and single-centered studies.^10,20,21,31^ We believe that this difference is mostly explained by the high proportion of patients with poor health status and without any treatment documented who were lost to follow up early and therefore censored in analysis.
  Initiation of guideline-concordant treatment was associated with improved survival for sub-classified NHL. For DLBCL, the most frequent NHL subtype in our cohort, the largest impact on survival of all variables studied was found for administration of at least 5 cycles of (R-)CHOP. In our study, DLBCL patients receiving CD20 antibodies in addition to CHOP appeared to have improved survival, but due to low patient numbers these findings were not statistically significant in our population-based setting. Findings from Malawi indicate that treatment including rituximab is feasible and cost-effective even in settings with high HIV prevalence (2-year OS: 55.5%).^17,34^ Similarly, the strongest impact for all NHL was administration of at least 5 cycles of any chemotherapy. These results have to be interpreted with caution since poor clinical status and subsequent early death were more likely found in the group with few cycles or no therapy. Nevertheless, our findings underline the necessity of subtype-directed and guideline-recommended treatment initiation and thorough administration of chemotherapy. Widely spread out-of-pocket expenditure inhibits both the continuation of chemotherapy as well as the adequate management of therapy side effects.^7,34,35^ Other reasons impeding completion of care include stigma of cancer disease^36,37^ and fear of therapy,^38^ travel distances to oncological centers,^39^ frequent stock out of chemotherapy,^40^ and supportive drugs.^22^
  The association between guideline-concordant approaches and improved survival is an encouraging result of our cohort study, but the effect of treatment of any kind was small compared to patients without any therapy documented. An observation from Uganda did not find benefit of treatment on survival.^22^ Though we were unable to find detailed data on side effects, we believe that infections and other toxicity-related side effects of chemo(immuno-)therapy overall reduce treatment benefits. Results of single-center NHL cohort studies show death from treatment-related complications in 9%-34% of patients.^18,20-22^ Therefore, there may be a need for patient stratification including dose reduction management and supportive care to offer tailored approaches in low-resource settings and eventually improve survival. To inform data-driven policy change regarding patient-centered provision of care, eg, further investigating the benefit of rituximab on survival, multicentre studies across the region should be conducted to address these global oncology challenges in SSA.^41^ In this context, it is important to note that our study confirms recent findings from SSA not showing the difference in survival between HIV-positive and -negative patients.^16-19^ Further, neither stage, ECOG PS, initiation of any treatment nor completion of at least 5 cycles of chemotherapy were influenced by HIV status in our cohort (Chi square test).
(3) Quality of care varied considerably within and between sites in terms of guideline-concordance and outcome. Addis Ababa, Nairobi, and Namibia had highest 1-year OS amounting up to 76.3%, whereas for Eldoret and Bulawayo it was as low as 37.5%. Proportion of patients diagnosed with NHL subtype ranged from 94.1% in Namibia to 8.3% in Maputo.^24^ Further, proportion of patients treated (any therapy) ranged from 71.6% (Nairobi) to 12.8% (Brazzaville), median number of cycles applied ranged from 6 to 1, and initiation of guideline-concordant treatment (including deviations) was found in some 30% of patients from Namibia, but in no patients from Maputo and Bamako. Radiotherapy was found in only 6.6% of all patients originating from 4 of 10 participating registries, matching availability of radiation at the time. This is in contrast to the actual need for radiotherapy that has been estimated up to 64% of NHL patients in low-and-middle income countries.^42^
  NHL survival trends in Western countries have tremendously improved in the last decades. For example, the 5-year-relative survival for US patients has continuously risen, from 56.3% in the period of 1990-1994^43^ to 73.2% in 2011-2017.^6^ Reasons include better understanding of lymphoma behavior, improved pathological and molecular diagnostics, a less harmful and more individualized therapy arsenal involving adapted polychemotherapy, monoclonal antibodies, targeted agents, bone marrow transplant, and, importantly, improved supportive care.
  Our data explore varying levels of the provision of adequate care in 11 oncological centers on population level and may serve as a baseline for targeting site-specific gaps. Generally, concerted efforts for long-lasting improvement of NHL survival in SSA should address enhancing diagnostic capacity,^12,24^ sustainable provision of guideline-recommended chemotherapy and elevation of oncological healthcare workforce,^44^ supportive,^45^ and palliative care.^46^ Prospective studies should examine the applicability of NCCN Harmonized Guidelines and focus on local shortcomings currently impeding significant advances in NHL care in the region.^7^

## Limitations and Strengths

The retrospective design of the study resulted in some limitations. First, imprecise staging, poor documentation, and early loss to follow up were frequent and have been reported from centers elsewhere.^10,22,47^ In 43.3% of patients it was not possible to acquire any additional information on diagnosis, treatment, or survival, limiting our report to registry baseline data. This might make some findings, eg, on clinical presentation, less precise than those from prospective, single-institution studies.^18-20,29^ It remains a subject of speculation whether patients not traceable have been facing particularly inadequate care, or even no treatment at all—or, quite the opposite, they left the registration area, eg, to seek more appropriate treatment. However, we assume that these patients are few since all of our study areas were major cities, usually providing the best cancer care in the country. We did include both public and private hospitals, and we estimate the proportion of affluent patients able to afford treatment is abroad rather small. Another possible reason for the high loss of follow-up is the problematic archiving system. Many study centers do not have well-established systems to document, trace and archive cases, and lack electronic databases. Nevertheless, it seems more likely that for a large share of the untraced cases, no therapy and therefore no medical records were initiated. In patients traced with incomplete therapy, we presume that a majority discontinued treatment due to a variety of reasons discussed above. In this sense, we consider the high share of loss to follow-up and the constricted diagnostic and therapeutic data not only a limiting factor of this study but also an important finding disclosing the concerning situation of NHL care in SSA.

Second, our survival data may reflect some selection bias. Overestimation of treatment effects is likely: (1) Reverse Kaplan-Meier analysis displayed that treatment was not selected at random, as patients with poor health status may not have been eligible for standard therapy, and some of these patients were censored early. (2) Patients with early deaths did not receive therapy, and (3) degree of guideline-concordance was only assessed during survival time and not before survival time started (immortal time bias, also known as survival bias).^48^ To reduce the overestimation of treatment effects and early deaths, we excluded patients surviving less than 1 month.^48^ For completion of eg, 6 cycles of CHOP, patients would have had to survive and remain in care for 4 months compared to our median follow-up of 6 months. However, follow-up data of our cohort was too poor to define a longer cutoff, and other cutoffs studied showed little differences in survival analysis. (4) Additionally, the random assignment of treatment could not be realized due to the observational design of the study.

Third, due to the shortage in diagnostic workup, sub-classification of almost 6 in 10 NHL was missing. Therefore, analysis of subtype-specific survival beyond OS was limited due to small patient numbers. We decided to hence limit in-depth calculations to the most frequent subtype, DLBCL.

There are important strengths to our study. First, we included a large population-based random sample of all NHL patients from 11 study centers involving both public and private institutions, not just those referred to specialist centers, and patients both with and without treatment. Second, the study involved a variety of countries in SSA, reflecting on a wide range of socioeconomic conditions and different health services in the region. Third, we were able to evaluate the impact of different treatment approaches—from guideline-concordant optimal therapy to none at all—on survival. This study is the first to create a link between NCCN Harmonized Guidelines and therapy actually received on the ground. It is, to our knowledge, the first population-based overview of cross-sectional and longitudinal data on therapy and outcome of NHL patients in real-world SSA.

## Conclusion

Advanced disease and considerable share of unclassified NHL reflect the lack of lymphoma awareness among healthcare personnel, poor referral systems, low pathological capacity, and high expenses of diagnosis that are hardly affordable for patients in low- and middle-income countries. Only a small proportion of patients from our cohort received NCCN guideline-concordant therapy, and these had better outcomes. Our results confirmed previous findings from SSA settings with high HIV prevalence that HIV in NHL appears to not be associated with worsened survival. For policymakers as well as institutions in SSA, our results can be an important baseline to plan, implement and measure targeted investments for improved outcomes of NHL patients. Cost-effective step-wise implementation of programs to allow guideline-concordant care should include: capacity-building for NHL subtyping, provision of therapeutic agents, supportive care and oncological workforce, fulfilling nursing requirements, and careful patient-centered care. Population-based cancer registries will facilitate monitoring these services over time.

## Supplementary Material

oyad157_suppl_Supplementary_Material

## Data Availability

Data supporting the findings in our study are available upon request. Requests will be evaluated by the AFCRN research committee. The data application process is outlined on the AFCRN website at http://afcrn.org/index.php/research/how-to-apply/76-research-collaborations.
