# Supplementary material for: Guideline Concordance of Treatment and Outcomes Among Adult Non-Hodgkin Lymphoma Patients in Sub-Saharan Africa: A Multinational, Population-Based Cohort
Source: Oncologist. 2023 Jun 27;28(11):e1017–30. doi: 10.1093/oncolo/oyad157 (PMC10628567; doi:10.1093/oncolo/oyad157)
Supplement: oyad157_suppl_Supplementary_Material [file oyad157_suppl_supplementary_material.docx]

**Supplement Table 1**: **Evaluation scheme for therapy initiation and completion**

“*”: derived from NCCN Harmonized Guidelines for Sub-Saharan Africa^10^

“#”: own definition of deviation from guidelines

R, rituximab; CHOP, cyclophosphamide, doxorubicin, vincristine, prednisone; COP, cyclophosphamide, vincristine, prednisone; CODOX-M, cyclophosphamide, cytarabine, vincristine, doxorubicin, methotrexate; IVAC, ifosfamide, etoposide, cytarabine; ECOG PS, Eastern Cooperative Oncology Group performance status; MALT lymphoma, mucosa-associated lymphoid tissue lymphoma

| **NHL subtype** | **Therapy evaluation** | **Therapy initiation - treatment modality** | **Patients (n)** | **Therapy completion - treatment modality** | **Patients (n)** |
| --- | --- | --- | --- | --- | --- |
| **Diffuse large B-cell lymphoma (n = 106)** | Guideline concordant | RCHOP* | 10 | At least 5 cycles of RCHOP* or (for stage I/II only) 3 cycles of RCHOP + Radiotherapy* | 10 |
|  | Deviation | CHOP* | 32 | At least 5 cycles of CHOP* or COP# or 4 cycles of RCHOP# or (for stage I/II only) 3 cycles of CHOP* or COP# + Radiotherapy | 30 |
|  | Any other therapy | Any other systemic therapy or radiotherapy only# | 14 | Less than 4 or unknown number of cycles of CHOP or less than 6 or unknown number of cycles of COP or any other systemic therapy or radiotherapy only | 26 |
|  | No therapy documented or not traced | No therapy including surgery only | 50 | No therapy including surgery only | 50 |
|  | **Total** |  | **106** |  | **106** |
| **NHL subtype** | **Therapy evaluation** | **Therapy initiation - treatment modality** | **Patients (n)** | **Therapy completion - treatment modality** | **Patients (n)** |
| **Burkitt lymphoma  (n = 13)** | Guideline concordant | CODOX-M / IVAC + Rituximab* | 0 | 4x CODOX-M / IVAC + Rituximab* | 0 |
|  | Deviation | CODOX-M / IVAC* or CHOP# | 3 | 4x CODOX-M / IVAC* or 5 cycles or more CHOP# | 3 |
|  | Any other therapy | Any other therapy# | 4 | Any other therapy# | 4 |
|  | No therapy documented or not traced | No therapy including surgery only | 6 | No therapy including surgery only | 6 |
|  | **Total** |  | **13** |  | **13** |
| **NHL subtype** | **Therapy evaluation** | **Therapy initiation - treatment modality** | **Patients (n)** | **Therapy completion - treatment modality** | **Patients (n)** |
| **Chronic lymphocytic leukemia / small lymphocytic lymphoma  (n = 40)** | Guideline concordant | Monotherapy Chlorambucil, Bendamustine or Cyclophosphamide +/- Rituximab* | 9 | Monotherapy Chlorambucil, Bendamustine or Cyclophosphamide +/- Rituximab*, any number of cycles | 9 |
|  | Observation guideline concordant | Watch and wait for early stage and good ECOG PS* | 0 | Watch and wait for early stage and good ECOG PS* | 0 |
|  | Deviation | Any polychemotherapy (CHOP or COP)# | 6 | Any polychemotherapy (CHOP or COP), any number of cycles# | 6 |
|  | Any other therapy | Any other systemic therapy# | 5 | Any other systemic therapy# | 5 |
|  | No therapy documented or not traced | No therapy including surgery only | 20 | No therapy including surgery only | 20 |
|  | **Total** |  | **40** |  | **40** |
| **NHL subtype** | **Therapy evaluation** | **Therapy initiation - treatment modality** | **Patients (n)** | **Therapy completion - treatment modality** | **Patients (n)** |
| **Follicular lymphoma  (n = 12)** | Therapy guideline concordant depending on stage, size, ECOG performance status and other criteria* | Radiotherapy* or guideline-recommended chemoimmunotherapy*: Bendamustine + rituximab or RCHOP or RCOP or R only; for elderly/infirm R+Chlorambucil or R+Cyclophosphamide or Chlorambucil only or Cyclophosphamide only | 1 | Completed radiotherapy (24-30 Gray)* or guideline-recommended chemoimmunotherapy, any number of cycles* | 1 |
|  | Observation guideline  concordant depending on stage, size, ECOG performance status and other criteria* | Observation only* | 1 | Watch and wait guideline concordant depending on stage, size and other criteria* | 1 |
|  | Deviation of guideline concordant therapy depending on stage, size, ECOG performance status and other criteria# | CHOP or other guideline-recommended chemotherapy regimen without rituximab# | 3 | CHOP or other guideline-recommended chemotherapy without rituximab, any number of cycles# | 3 |
|  | Any other therapy than guideline concordant therapy or deviation depending on stage, size and other criteria# | Any other non guideline-recommended systemic therapy# | 2 | Any other non guideline-recommended systemic therapy# | 2 |
|  | No therapy documented or not traced | No therapy including surgery only | 5 | No therapy including surgery only | 5 |
|  | **Total** |  | **12** |  | **12** |
| **NHL subtype** | **Therapy evaluation** | **Therapy initiation - treatment modality** | **Patients (n)** | **Therapy completion - treatment modality** | **Patients (n)** |
| **Marginal zone Lymphoma  (n = 7)** | Guideline concordant | Bendamustine + Rituximab*; RCHOP*; RCOP*; Monotherapy for elderly or infirm; antibiotic therapy for gastric MALT lymphoma*; surgery for early stage nongastric MALT lymphoma* | 0 | Bendamustine + Rituximab*; RCHOP*; RCOP*; Monotherapy for elderly or infirm, any number of cycles; antibiotic therapy for gastric MALT lymphoma*; surgery for early stage nongastric MALT lymphoma* | 0 |
|  | Deviation | CHOP# | 3 | CHOP, any number of cycles# | 3 |
|  | Any other therapy | Any other systemic therapy# | 0 | Any other therapy# | 0 |
|  | No therapy documented or not traced | No therapy including surgery only (excluding for nongastric early stage MALT lymphoma) | 4 | No therapy including surgery only | 4 |
|  | **Total** |  | **7** |  | **7** |
| **NHL subtype** | **Therapy evaluation** | **Therapy initiation - treatment modality** | **Patients (n)** | **Therapy completion - treatment modality** | **Patients (n)** |
| **Lymphoplasmacytic lymphoma  (n = 2)** | Any other therapy | Unknown chemotherapy# | 1 | Unknown chemotherapy# | 1 |
|  | No therapy documented or not traced | No therapy including surgery only | 1 | No therapy including surgery only | 1 |
|  | **Total** |  | **2** |  | **2** |
|  | **Therapy evaluation** | **Therapy initiation - treatment modality** | **Patients (n)** | **Therapy completion - treatment modality** | **Patients (n)** |
| **Other subtypes without NCCN guidelines harmonized for Sub-Saharan Africa available  (n = 37)** | More intensive therapy | Any polychemotherapy | 14 | 5 or more cycles of any therapy | 9 |
|  | Less intensive therapy | Any other therapy | 0 | less than 5 cycles or unknown number of cycles | 5 |
|  | No therapy documented or not traced | No therapy including surgery only | 23 | No therapy including surgery only | 23 |
|  | **Total** |  | **37** |  | **37** |
| **Unclassified lymphoma  (n = 299** | More intensive therapy | Any polychemotherapy | 60 | 5 or more cycles of any therapy | 39 |
|  | Less intensive therapy | Any other therapy | 27 | less than 5 cycles or unknown number of cycles | 48 |
|  | No therapy documented or not traced | No therapy including surgery only | 212 | No therapy including surgery only | 212 |
|  | **Total** |  | **299** |  | **299** |

**Supplement Table 2: Population-based cancer registries (PBCR) and study population characteristics.**

| **PBCR  (years observed)** | **Population covered by PBCR^28^** | **Patients registered in PBCR during years observed** | **Population-based sample (n (% of patients registered in PBCRs during years observed))** | **Patients excluded (n (% of population-based sample))** | **Total cohort (n)** | **Patients traced (n (% of total cohort)** | **Patients initiating any therapy (n (% of total cohort)** | **Cycles of chemo received (median, IQR)** | **Patients completing 5 or more cycles of any chemotherapy (n (% of total cohort)** |
| --- | --- | --- | --- | --- | --- | --- | --- | --- | --- |
| Abidjan  (2012-2013) | 4.402.949 | 112 | 59 (52.7) | 16 (27.1) | 43 | 30 (69.8) | 12 (27.3) | 6 (3-8) | 5 (11.4) |
| Addis Ababa (2012&2014) | 3.050.000 | 103 | 86 (83.5) | 16 (18.6) | 70 | 33 (47.1) | 40 (57.1) | 6 (2-7) | 12 (17.1) |
| Bamako  (2012-2013) | 1.810.366 | 61 | 60 (98.4) | 7 (11.7) | 53 | 20 (37.8) | 8 (15.4) | 1 (1-7) | 2 (3.8) |
| Brazzaville (2011-2014) | 1.549.693 | 42 | 42 (100) | 3 (7.1) | 39 | 6 (15.4) | 5 (12.8) | 4 (2-6) | 1 (2.6) |
| Bulawayo (2012-2013) | 653.000 | 198 | 60 (30.3) | 7 (11.7) | 53 | 36 (67.9) | 18 (34.0) | 4 (2-8) | 6 (11.3) |
| Cotonou (2013-2014) | 678.874 | 8 | 8 (100) | 7 (87.5) | 1 | 1 (100) | 0 | n/a | 0 |
| Eldoret  (2012-2013) | 894.179 | 68 | 60 (88.2) | 3 (5.0) | 57 | 21 (36.8) | 12 (21.1) | 5 (2-6) | 7 (12.3) |
| Kampala (2012-2013) | 2.010.000 | 94 | 59 (62.8) | 4 (6.8) | 55 | 40 (72.7) | 17 (30.9) | 6 (4-6) | 9 (16.4) |
| Maputo (2014-2015) | 1.225.868 | 25 | 25 (100) | 1 (4.0) | 24 | 17 (70.8) | 12 (50) | 4 (2-6) | 4 (16.7) |
| Nairobi  (2012-2013) | 3.138.369 | 196 | 60 (30.6) | 7 (11.7) | 53 | 44 (83.0) | 38 (71.7) | 6 (6-8) | 22 (41.5) |
| Namibia (2012-2013) | 2.104.900 | 161 | 80 (49.7) | 12 (15.0) | 68 | 45 (66.2) | 33 (48.5) | 6 (4-6) | 23 (33.8) |
| **11 PBCRs (2011-2015)** | **21.518.198** | **1.068** | **599 (56.1)** | **83 (13.9)** | **516** | **293 (56.8)** | 195 (37.8) | 6 (3-6) | 91 (17.6) |

**Supplement Table 3. Demographics, diagnostic modality and clinical presentation.**

Unknown information were not included in calculating proportions. *: Information for traced patients (n=293) available only. Taken from:

Mezger NCS, Feuchtner J, Griesel M, et al. Clinical presentation and diagnosis of adult patients with non-Hodgkin lymphoma in Sub-Saharan Africa. *British journal of haematology*. 2020;190(2):209-221. Published March 17, 2020.

|  |  | **Total cohort (n (%))** |
| --- | --- | --- |
| **Sex** | Female | 224 (43.4) |
|  | Male | 292 (56.6) |
| **Age** | Median | 45 (15-93) |
|  | 15-39 | 202 (39.1) |
|  | 40-59 | 204 (39.5) |
|  | 60+ | 110 (22.3) |
| **Diagnostic modality** | Histology | 366 (76.2) |
|  | FNAC | 83 (17.3) |
|  | Clinical | 31 (6.5) |
|  | Unknown | 36 |
| **Primary site involved** | Nodal | 385 (81.4) |
|  | Extranodal | 88 (18.6) |
|  | Unknown | 43 |
| **B symptoms*** | No | 19 (20.7) |
|  | Yes | 73 (79.3) |
|  | Unknown | 424 |
| **ECOG Performance Score*** | 0 or 1 | 51 (38.6) |
|  | 2 and above | 81 (61.4) |
|  | Unknown | 384 |
| **Stage*** | Early | 48 (27.0) |
|  | Advanced | 130 (73.0) |
|  | Unknown | 338 |
| **HIV*** | Negative | 57 (37.0) |
|  | Positive | 97 (63.0) |
|  | Unknown | 362 |
| **Imaging*** | CT/MRI/Bone Scan | 36 (12.3) |
|  | X-Ray and/or US | 83 (28.3) |
|  | None | 174 (59.4) |
|  | Unknown | 223 |

**Supplement Table 4: Morphological classification of NHL**

Stratified by sub-classified high grade B-cell NHL, sub-classified low-grade B-cell NHL, sub-classified T-cell NHL, otherwise sub-classified NHL, unclassified, graded NHL and unclassified lymphoma. Taken from:

Mezger NCS, Feuchtner J, Griesel M, et al. Clinical presentation and diagnosis of adult patients with non-Hodgkin lymphoma in Sub-Saharan Africa. *British journal of haematology*. 2020;190(2):209-221. Published March 17, 2020.

“+”: percentage of population-based cohort. “*”: percentage of all sub-classified NHL. “+”: percentage of total cohort. “#”: NCCN Harmonized Guidelines available.^10^ „†“: No NCCN Harmonized Guidelines available.^10^

| **Lymphoma classification** | | **ICD-O morphology codes** | **Patients (n (%))** |
| --- | --- | --- | --- |
| **All sub-classified NHL** | |  | **217 (42.1)+** |
| **Sub-classified high-grade B-cell NHL** | |  | **121 (55.8)*** |
|  | Diffuse large B-cell# | 9680, 9684 | 106 (48.8)* |
|  | Burkitt# | 9687 | 13 (6.0)* |
|  | Precursor lymphoblastic B-cell† | 9728 | 1 (0.5)* |
|  | Plasmablastic† | 9735 | 1 (0.5)* |
| **Sub-classified low-grade B-cell NHL** | |  | **64 (29.5)*** |
|  | CLL/SLL# | 9823, 9670 | 40 (18.4) |
|  | Follicular# | 9690, 9695, 9698 | 12 (5.5)* |
|  | Marginal zone# | 9710, 9689, 9699 | 7 (3.2)* |
|  | Mantle cell† | 9673 | 3 (1.4)* |
|  | Lymphoplasmacytic# | 9671 | 2 (0.9)* |
| **Sub-classified T-cell NHL** | |  | **15 (6.9)*** |
|  | Anaplastic large T-/Null-cell† | 9714 | 5 (2.3)* |
|  | Mature T-cell, NOS† | 9702 | 3 (1.4)* |
|  | Mycosis fungoides† | 9700 | 3 (1.4)* |
|  | Angioimmunoblastic T-cell† | 9705 | 1 (0.5)* |
|  | Precursor T-cell lymphoblastic† | 9729 | 1 (0.5)* |
|  | Natural Killer/T-cell† | 9719 | 1 (0.5)* |
|  | Sezary syndrome† | 9701 | 1 (0.5)* |
| **Otherwise sub-classified NHL** | |  | **17 (7.8)*** |
|  | Composite Hodgkin and Non-Hodgkin lymphoma† | 9596 | 8 (3.7)* |
|  | Precursor cell lymphoblastic, unknown cellular lineage† | 9727 | 8 (3.7)* |
|  | Disseminated Langerhans cell histiocytosis† | 9754 | 1 (0.5)* |
| **All unclassified lymphoma** | |  | **299 (57.9)+** |
| **Unclassified, graded NHL** | |  | **42 (8.1)+** |
|  | High-grade B-cell, NOS# | 9591 | 4 (0.8)+ |
|  | Low-grade B-cell, NOS† | 9591 | 2 (0.4)+ |
|  | High-grade, unknown cellular lineage, NOS† | 9591 | 24 (4.7)+ |
|  | Low-grade, unknown cellular lineage, NOS† | 9591 | 12 (2.3)+ |
| **Unclassified NHL, not graded** | |  | **257 (48.6)+** |
|  | Unclassified NHL, NOS† | 9591 | 165 (32.0)+ |
|  | Unclassified NHL or HL, NOS† | 9590 | 92 (17.8)+ |
| **Total cohort** | |  | **516 (100)+** |

**Supplement Table 5: Detailed therapy modalities.**

CHOP, cyclophosphamide, doxorubicin, vincristine, prednisone; COP, cyclophosphamide, vincristine, prednisone; PCT, polychemotherapy; RCHOP, rituximab, cyclophosphamide, doxorubicin, vincristine, prednisone; MTX, methotrexate; RCOP, rituximab, cyclophosphamide, vincristine, prednisone; ABV, doxorubicin, bleomycin, vincristine; CEVP, cyclophosphamide, etoposide, vincristine, prednisone; CODOX-M, cyclophosphamide, cytarabine, vincristine, doxorubicin, methotrexate; IVAC, ifosfamide, etoposide, cytarabine; ICE, ifosfamide, carboplatin, etoposide; ABVP, doxorubicin, bleomycin, vincristine, prednisone; DEP, cisplatin, etoposide, prednisone; NOS, not otherwise specified: RT, radiotherapy

| **Chemo regimen** | **Patients (n)** | **% of all receiving chemo** | **Chemotherapy regimen, # of cycles** | **Patients (n)** | **% of all receiving chemo** | **median # of cycles** | **range of cycles** |
| --- | --- | --- | --- | --- | --- | --- | --- |
| **CHOP and derivatives** | **116** | **62** | CHOP, 5 or more | 56 | 29.9 | 6 | 5 to 9 |
|  |  |  | CHOP, less than 5 | 30 | 16.0 | 2 | 1 to 4 |
|  |  |  | CHOP, unknown # of cycles | 11 | 5.9 | n/a | n/a |
|  |  |  | CHOP+Bleomycin, 6 | 1 | 0.5 | n/a | n/a |
|  |  |  | CHOP+Bleomycin, 3 | 1 | 0.5 | n/a | n/a |
|  |  |  | CHOP+Etoposide, 1 | 1 | 0.5 | n/a | n/a |
|  |  |  | RCHOP, 5 or more | 13 | 7.0 | 6 | 6 to 8 |
|  |  |  | RCHOP, less than 5 | 2 | 1.1 | 4 | 4 |
|  |  |  | RCHOP, unknown # of cycles | 1 | 0.5 | n/a | n/a |
| **COP and derivatives** | **20** | **10.7** | COP, 5 or more | 9 | 4.8 | 6 | 5 to 11 |
|  |  |  | COP, less than 5 | 6 | 3.2 | 1.5 | 1 to 2 |
|  |  |  | COP, unknown # of cycles | 1 | 0.5 | n/a | n/a |
|  |  |  | COP + MTX, 2 | 1 | 0.5 | n/a | n/a |
|  |  |  | COP+Etoposide, less than 5 | 2 | 1.1 | 1.5 | 1 to 2 |
|  |  |  | RCOP, 9 | 1 | 0.5 | n/a |  |
| **Other PCT regimen** | **8** | **4.3** | ABV, 4 | 1 | 0.5 | n/a | n/a |
|  |  |  | CEVP, 5 or more | 1 | 0.5 | n/a | n/a |
|  |  |  | CODOX-M/IVAC, 4 | 1 | 0.5 | n/a | n/a |
|  |  |  | ICE, 1 | 1 | 0.5 | n/a | n/a |
|  |  |  | Cisplatin+Paclitaxel, 3 | 1 | 0.5 | n/a | n/a |
|  |  |  | ABVP, 1 | 1 | 0.5 | n/a | n/a |
|  |  |  | DEP, 3 | 1 | 0.5 | n/a | n/a |
|  |  |  | Rituximab and Bendamustine, 4 | 1 | 0.5 | n/a | n/a |
| **Monotherapy regimen** | **15** | **8** | Chlorambucil, less than 5 | 2 | 1.1 | 2.5 | 1 to 4 |
|  |  |  | Chlorambucil, unknown # of cycles | 6 | 3.2 | n/a | n/a |
|  |  |  | Vincristine and Prednisone, 6 | 2 | 1.1 | n/a | n/a |
|  |  |  | Vincristine and Prednisone, 2 | 1 | 0.5 | n/a | n/a |
|  |  |  | Rituximab, 4 | 2 | 1.1 | n/a | n/a |
|  |  |  | Cyclophosphamide and Prednisone, unknown # of cycles | 1 | 0.5 | n/a | n/a |
|  |  |  | Gemcitabine, 4 | 1 | 0.5 | n/a | n/a |
| **Unknown chemo regimen** | **28** | **15** | Chemo unknown, 5 or more | 5 | 2.7 | 6 | 5 to 8 |
|  |  |  | Chemo unknown, less than 5 | 2 | 1.1 | 3 | 2 to 4 |
|  |  |  | Chemo unknown, unknown # of cycles | 21 | 11.2 | n/a | n/a |
|  |  |  | **Total** | **187** | **100** | **6** | **1 to 11** |
| **Radiotherapy** |  |  | **Gray** | **n** | **% of all receiving RT** |  | |
|  |  |  | 30 Gray and above | 17 | 50 |  |  |
|  |  |  | Below 30 Gray | 7 | 20.6 |  |  |
|  |  |  | Unknown | 10 | 29.4 |  |  |
|  |  |  | **Total** | **34** | **100** |  |  |
| **Lymphoma-directed Surgery** |  |  | **Surgery type** | **n** | **% of all receiving surgery** |  | |
|  |  |  | (Partial) Lymphoma resection, NOS | 8 | 28.6 |  |  |
|  |  |  | Hemicolectomy | 5 | 17.9 |  |  |
|  |  |  | Splenectomy | 5 | 17.9 |  |  |
|  |  |  | Abdominal surgery, NOS | 3 | 10.7 |  |  |
|  |  |  | Unknown | 7 | 25 |  |  |
|  |  |  | **Total** | **28** | **100** |  |  |

**Supplement Table 6: Overall survival by Kaplan-Meier estimates for total population-based cohort stratified by registry**

OS, overall survival; std, standard deviation

Cotonou was excluded from table and figure due to small sample size

|  |  |  | **1 year** | | | **2 years** | | | **3 years** | | | **5 years** | | |
| --- | --- | --- | --- | --- | --- | --- | --- | --- | --- | --- | --- | --- | --- | --- |
| **Registry** | **Cohort (n)** | **Median survival in months** | **OS** | **std** | **No at risk** | **OS** | **std** | **No at risk** | **OS** | **std** | **No at risk**  **(3 years)** | **OS** | **std** | **No at risk** |
| Addis | 70 | 25 | **76.3%** | 0.065* | 25 | 53.5% | 0.091* | 11 | 42.8% | 0.100* | 5 | n/a | n/a | n/a |
| Nairobi | 53 | 33 | **71.9%** | 0.070 | 25 | 50.4% | 0.084 | 15 | 43.2% | 0.086 | 12 | 43.2% | 0.086 | 2 |
| Namibia | 68 | n/a | **70.6%** | 0.071 | 23 | 63.9% | 0.079 | 19 | 53.8% | 0.085 | 16 | 50.2% | 0.087 | 7 |
| Maputo | 24 | n/a | **68.1%** | 0.132 | 7 | 54.4% | 0.161 | 0 | n/a | n/a | n/a | n/a | n/a | n/a |
| **All** | **516** | **20** | **61.2%** | **0.030** | **127** | **45.1%** | **0.034** | **70** | **37.3%** | **0.036** | **50** | **35.4** | **0.036** | **31** |
| Brazzaville | 39 | 14 | **59.9%** | 0.124* | 3 | 30.0% | 0.221* | 1 | 0% | 0 | 0 | n/a | n/a | n/a |
| Abidjan | 43 | 18 | **60.0%** | 0.115* | 8 | 45.0% | 0.126* | 6 | 45.0% | 0.126* | 5 | 36.0% | 0.129* | 1 |
| Kampala | 55 | 15 | **57.1%** | 0.089 | 16 | 33.0% | 0.091 | 8 | 28.9% | 0.088 | 7 | n/a | n/a | n/a |
| Bamako | 53 | 23 | **53.7%** | 0.141* | 6 | 43.0% | 0.148* | 4 | 43.0% | 0.148* | 3 | n/a | n/a | n/a |
| Eldoret | 57 | 9 | **40.1%** | 0.108 | 7 | 17.8% | 0.101 | 2 | 17.8% | 0.101 | 2 | 17.8% | 0.101 | 1 |
| Bulawayo | 53 | 9 | **37.5%** | 0.108 | 6 | 31.2% | 0.106 | 5 | 12.5% | 0.102 | 1 | n/a | n/a | n/a |


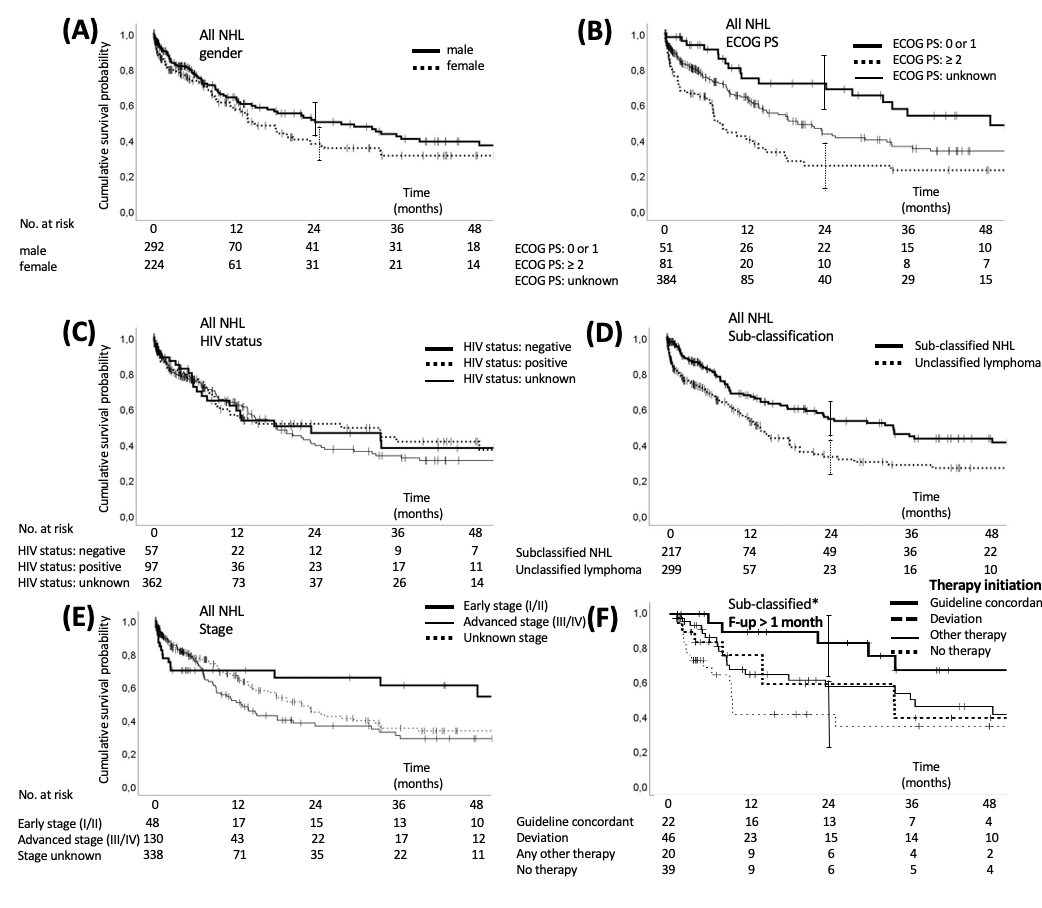


**Supplement Figure 1**: **Kaplan Meier estimates.**

Survival of all NHL stratified by gender (A); ECOG performance status (B); HIV status (C); sub-classification (D); stage (E); therapy initiation with respect to concordance with guidelines for all patients with sub-classified NHL and guidelines available (F)

**Supplement Table 7: Univariable Cox regression**

Patients with follow-up above 30 days and diagnosis of any NHL (n=296) diffuse large B-cell lymphoma (n=74). Cox proportional hazard univariable and multivariable analysis for overall survival.

| **All NHL with follow-up >30 days (n = 296** **)** | 121 events | Unadjusted hazard ratio | Lower 95% CI | Upper 95% CI | p |
| --- | --- | --- | --- | --- | --- |
| **Sex** | Male (reference) |  |  |  |  |
|  | Female | 1,151 | 0,805 | 1,644 | 0,441 |
| **Age** | 15-39 (reference) |  |  |  |  |
|  | 40-59 | 0,73 | 0,447 | 1,194 | 0,21 |
|  | 60+ | 0,768 | 0,479 | 1,229 | 0,271 |
| **ECOG PS** | 0 or 1 (reference) |  |  |  |  |
|  | 2 or worse | 2,498 | 1,354 | 4,609 | 0,003 |
|  | Unknown | 1,636 | 0,952 | 2,81 | 0,075 |
| **Stage** | Early (I/II) (reference) |  |  |  |  |
|  | Advanced (III/IV) | 3,099 | 1,403 | 6,845 | 0,005 |
|  | Unknown | 2,387 | 1,089 | 5,231 | 0,03 |
| **HIV status** | Negative (reference) |  |  |  |  |
|  | Positive | 0,878 | 0,492 | 1,566 | 0,66 |
|  | Unknown | 1,145 | 0,691 | 1,898 | 0,599 |
| **B symptoms** | No B symptoms (reference) |  |  |  |  |
|  | B symptoms | 3,883 | 1,165 | 12,938 | 0,027 |
|  | Unknown | 3,285 | 1,038 | 10,394 | 0,043 |
| **Sub-classification** | Sub-classified (reference) |  |  |  |  |
|  | Unclassified | 1,293 | 0,903 | 1,853 | 0,161 |
| **Cycles of any chemo(immuno-)therapy regimen received;** | 5 or more cycles (reference) |  |  |  |  |
|  | Less than 5 cycles | 2,693 | 1,674 | 4,335 | 0.000 |
|  | Not given | 2,313 | 1,45 | 3,689 | 0.000 |
| **All DLBCL with follow-up >30 days (n = 74)** | 27 events | Unadjusted hazard ratio | Lower 95% CI | Upper 95% CI | p |
| **Sex** | Male (reference) |  |  |  |  |
|  | Female | 1,385 | 0,64 | 2,996 | 0,408 |
| **Age** | 15-39 (reference) |  |  |  |  |
|  | 40-59 | 0,796 | 0,329 | 1,927 | 0,613 |
|  | 60+ | 3,041 | 1,123 | 8,234 | 0,029 |
| **ECOG PS** | 0 or 1 (reference) |  |  |  |  |
|  | 2 or worse | 2,026 | 0,64 | 6,415 | 0,23 |
|  | Unknown | 2,095 | 0,757 | 5,793 | 0,154 |
| **Stage** | Early (I/II) (reference) |  |  |  |  |
|  | Advanced (III/IV) | 1,22 | 0,364 | 4,088 | 0,747 |
|  | Unknown | 3,152 | 1,032 | 9,625 | 0,044 |
| **HIV status** | Negative (reference) |  |  |  |  |
|  | Positive | 1,012 | 0,317 | 3,23 | 0,984 |
|  | Unknown | 1,788 | 0,583 | 5,49 | 0,31 |
| **B symptoms** | No B symptoms (reference) |  |  |  |  |
|  | B symptoms | 1,396 | 0,252 | 7,715 | 0,702 |
|  | Unknown | 2,299 | 0,534 | 9,903 | 0,264 |
| **Guideline concordance** | Guideline concordant or deviation (reference) |  |  |  |  |
|  | Any other therapy | 2,781 | 1,301 | 5,944 | 0,008 |
|  | no therapy | 2,908 | 1,403 | 10,892 | 0,001 |
